# The platelet isoform of phosphofructokinase contributes to metabolic reprogramming and maintains cell proliferation in clear cell renal cell carcinoma

**DOI:** 10.18632/oncotarget.8382

**Published:** 2016-03-25

**Authors:** Jun Wang, Ping Zhang, Jie Zhong, Mingyue Tan, Jifu Ge, Le Tao, Yakui Li, Yemin Zhu, Lifang Wu, Jianxin Qiu, Xuemei Tong

**Affiliations:** ^1^ Department of Urology, Shanghai General Hospital, Shanghai Jiao Tong University School of Medicine, Shanghai, China; ^2^ Department of Biochemistry and Molecular Cell Biology, Shanghai Key Laboratory for Tumor Microenvironment and Inflammation, Shanghai Jiao Tong University School of Medicine, Shanghai, China

**Keywords:** PFKP, aerobic glycolysis, proliferation, clear cell renal cell carcinoma

## Abstract

Metabolic alterations underlying clear cell renal cell carcinoma (ccRCC) progression include aerobic glycolysis, increased pentose phosphate pathway activity and reduced oxidative phosphorylation. Phosphofructokinase (PFK), a key enzyme of the glycolytic pathway, has L, M, and P isoforms with different tissue distributions. The mRNA level of the platelet isoform of phosphofructokinase (PFKP) is reported to be up-regulated in ccRCC patients. However, it remains unclear whether PFKP plays an important role in promoting aerobic glycolysis and macromolecular biosynthesis to support cell proliferation in ccRCC. Here we found that the up-regulated PFKP became the predominant isoform of PFK in human ccRCC. Suppression of PFKP not only impaired cell proliferation by inducing cell cycle arrest and apoptosis, but also led to decreased glycolysis, pentose phosphate pathway and nucleotide biosynthesis, accompanied by activated tricarboxylic acid cycle in ccRCC cells. Moreover, we found that p53 activation contributed to cell proliferation and metabolic defects induced by PFKP knockdown in ccRCC cells. Furthermore, suppression of PFKP led to reduced ccRCC tumor growth *in vivo*. Our data indicate that PFKP not only is required for metabolic reprogramming and maintaining cell proliferation, but also may provide us with a valid target for anti-renal cancer pharmaceutical agents.

## INTRODUCTION

Clear cell renal cell carcinoma (ccRCC) often displays dysregulated glucose or glutamine metabolism with alterations in oxygen, energy and nutrient sensing [1–3]. Unlike normal kidney tissues, ccRCC is characterized by aerobic glycolysis or Warburg effect, increased dependence on the pentose phosphate pathway, reduced tricarboxylic acid (TCA) cycle activity, augmented glutamine transport, fatty acid accumulation and low AMP-activated protein kinase (AMPK) activity [1–3]. Genes typically mutated in ccRCC including *VHL*, *MET*, *FLCN*, *FH*, *SDH*, *TSC1* and *TSC2* have been found to play a critical role in regulating cellular metabolic processes [4, 5]. Therefore, a deep understanding of the metabolic abnormalities underlying ccRCC initiation and progression will provide us with new opportunities for developing novel therapeutic strategies for the disease.

Aerobic glycolysis is the basis for other metabolic features of ccRCC because some glucose can be diverted from oxidative phosphorylation towards synthesizing macromolecular precursors, such as acetyl-CoA for fatty acids, glycolytic intermediates for nonessential amino acids, and ribose-5-phosphate for nucleotides [2, 6]. This way ccRCC can obtain adequate carbon, nitrogen, free energy, and reducing equivalents to support cell growth and division. The reliance of ccRCC on aerobic glycolysis has been mostly attributed to mutations in the VHL/HIF pathway and subsequent up-regulation of HIF-target genes in glucose metabolism such as glucose transporter 1 (GLUT1), phosphoglycerate kinase 1 (PGK1), lactate dehydrogenase A (LDHA) and pyruvate dehydrogenase kinase 1 (PDK1) [7, 8]. Moreover, ccRCC shows high glucose-6-phosphate-dehydrogenase (G6PD) and transketolase activity, which are key enzymes for the oxidative and non-oxidative branches of the pentose phosphate pathway, respectively [9, 10]. The pentose phosphate pathway provides both ribose-5-phosphate for nucleotide biosynthesis and NADPH for promoting reductive processes including fatty acids and cholesterol biosynthesis. Recent findings reveal that fructose-1,6-bisphosphatase 1 (FBP1), a gluconeogenic enzyme that hydrolyzes fructose 1,6-bisphosphate to fructose 6-phosphate, is down-regulated in ccRCC [11]. FBP1 depletion increases glycolytic flux in an enzyme activity dependent and independent manner [11]. Interestingly, the liver isoform of the critical glycolytic enzyme phosphofructokinase (PFKL), which catalyzes the reverse reaction of FBP1, is expressed at equal levels in ccRCC and control kidney tissues [11].

Phosphofructokinase (PFK), catalyzing the formation of fructose 1,6-bisphosphate and ADP from fructose 6-phosphate and ATP, is a rate-controlling enzyme of the glycolytic pathway. Fructose 1,6-bisphosphate, the product of PFK, can function as a signal molecule to activate liver pyruvate kinase, inhibit mitochondrial oxidative phosphorylation, and regulate reactive oxygen species levels [12–16]. Mammalian PFK is a homo- or hetero-tetramer of L, M, and P isoforms [17, 18]. In normal tissues, PFKL is mainly expressed in liver and kidney while skeletal muscle and platelets have mostly PFKM and PFKP, respectively [17, 18]. In tumors and cancer cell lines, P or L, or both isoforms are most abundant [19, 20]. Transcriptomic studies have identified PFKP as a significantly up-regulated glycolytic gene in ccRCC patients [10, 21, 22]. However, it remains unclear whether PFKP is the predominant isoform of PFK in ccRCC and how PFKP plays a role in regulating metabolism and cell proliferation in ccRCC.

In this report, we first showed that PFKP was the predominant isoform of PFK in human ccRCC tissues. Next we found that PFKP knockdown inhibited cell proliferation, induced apoptosis and attenuated tumorigenic capacity partially through the p53 pathway in renal cancer cells. Moreover, PFKP knockdown resulted in decreased aerobic glycolysis, increased oxygen consumption and reduced pentose phosphate pathway as well as *de novo* nucleotide biosynthesis. Our results demonstrate that PFKP, the predominant PFK isoform in ccRCC, plays a key role in promoting aerobic glycolysis and anabolism as well as suppressing p53 activity to maintain rapid proliferation.

## RESULTS

### PFKP is up-regulated in human ccRCC

**Figure 1:**
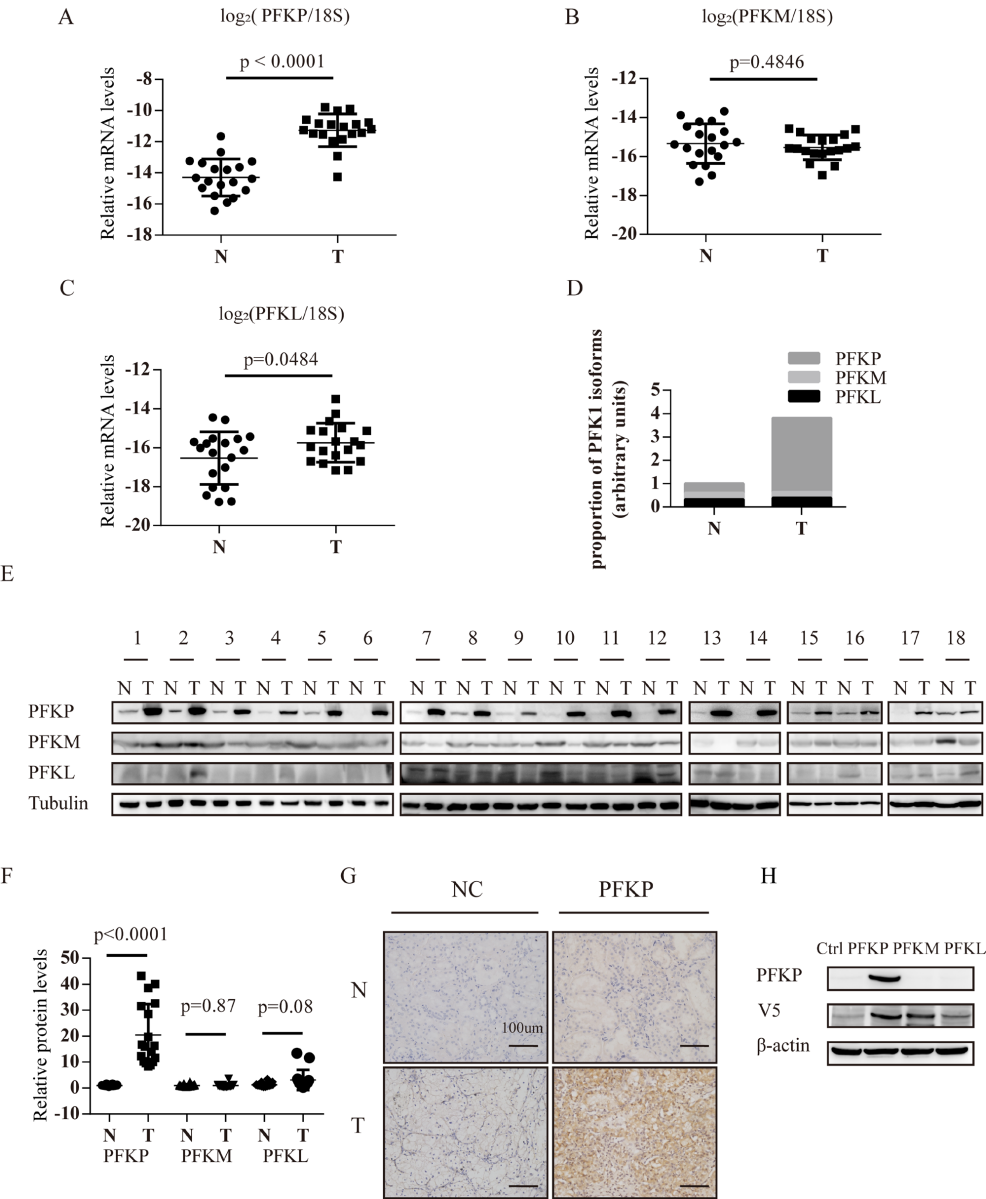
PFKP mRNA and protein is up-regulated in human ccRCC tissues **A.**-**C.** Real time PCR analysis for PFKP (A), PFKM (B) and PFKL (C) mRNA levels in human ccRCC tissue samples (T) and adjacent non-malignant kidney tissues (N) (*n* = 19). 18S ribosomal RNA serves as an internal control. (D) Bar graph for average percentage of PFKP, PFKM and PFKL mRNA in 18 human ccRCC tissue samples (T) and 18 adjacent non-malignant kidney tissues (N) based on absolute quantification PCR analysis. **E.** Western blot for protein extracts from human ccRCC tissue samples (T) and adjacent non-malignant kidney tissues (N) (*n* = 18). **F.** Quantification of western blot for PFKP, PFKM, PFKL using Gel-Pro analyzer 4. **G.** Immunohistochemistry analysis for ccRCC and non-malignant kidney tissue samples using either normal serum (NC) or the anti-PFKP antibody. **H.** Western blot for protein extracts of 293T cells transiently transfected with the control vector or plasmids containing V5-tagged PFKP, PFKM or PFKL cDNA.

To validate up-regulation of PFKP at the transcriptional level in ccRCC, we compared PFKP mRNA levels in 19 ccRCC tumor and 19 adjacent non-malignant kidney tissue samples using quantitative PCR. We found that PFKP was consistently up-regulated in ccRCC tumor samples (Figure 1A). We also found that mRNA levels of PFKM were similar in ccRCC and non-malignant samples while PFKL was slightly up-regulated in tumor samples (Figure 1B and 1C). We next estimated relative abundance of mRNAs of the three PFK isoforms in ccRCC and adjacent non-malignant kidney tissues using quantitative PCR. Percentage of PFKP, PFKM and PFKL mRNAs was 39.7%, 32.1% and 28.2% in non-malignant kidney tissues, respectively (Figure 1D). Interestingly, PFKP was up-regulated and became the predominant isoform (83.2%) in ccRCC (Figure 1A and 1D).

We further compared PFKP, PFKM and PFKL protein levels in ccRCC and adjacent non-malignant kidney tissue samples using Western blot analysis and immunohistochemistry (Figure 1E and 1G). Quantifications of Western blot were summarized in Figure 1F. The specificity of the anti-PFKP antibody was verified by performing Western blot analysis for protein extract of 293T cells ectopically expressing PFKP, PFKM or PFKL cDNA (Figure 1H). The protein level of PFKP was consistently higher in 18 ccRCC samples compared with their adjacent non-malignant kidney tissues (Figure 1E and 1F), while PFKM and PFKL proteins show comparable levels between normal and cancer groups (Figure 1E and 1F). Immunohistochemistry using the anti-PFKP antibody showed that PFKP was abundantly expressed in ccRCC cells whereas PFKP expression was hardly detectable in non-malignant kidney tissues (Figure 1G).

### PFKP is required for cell proliferation in kidney cancer cell lines

**Figure 2:**
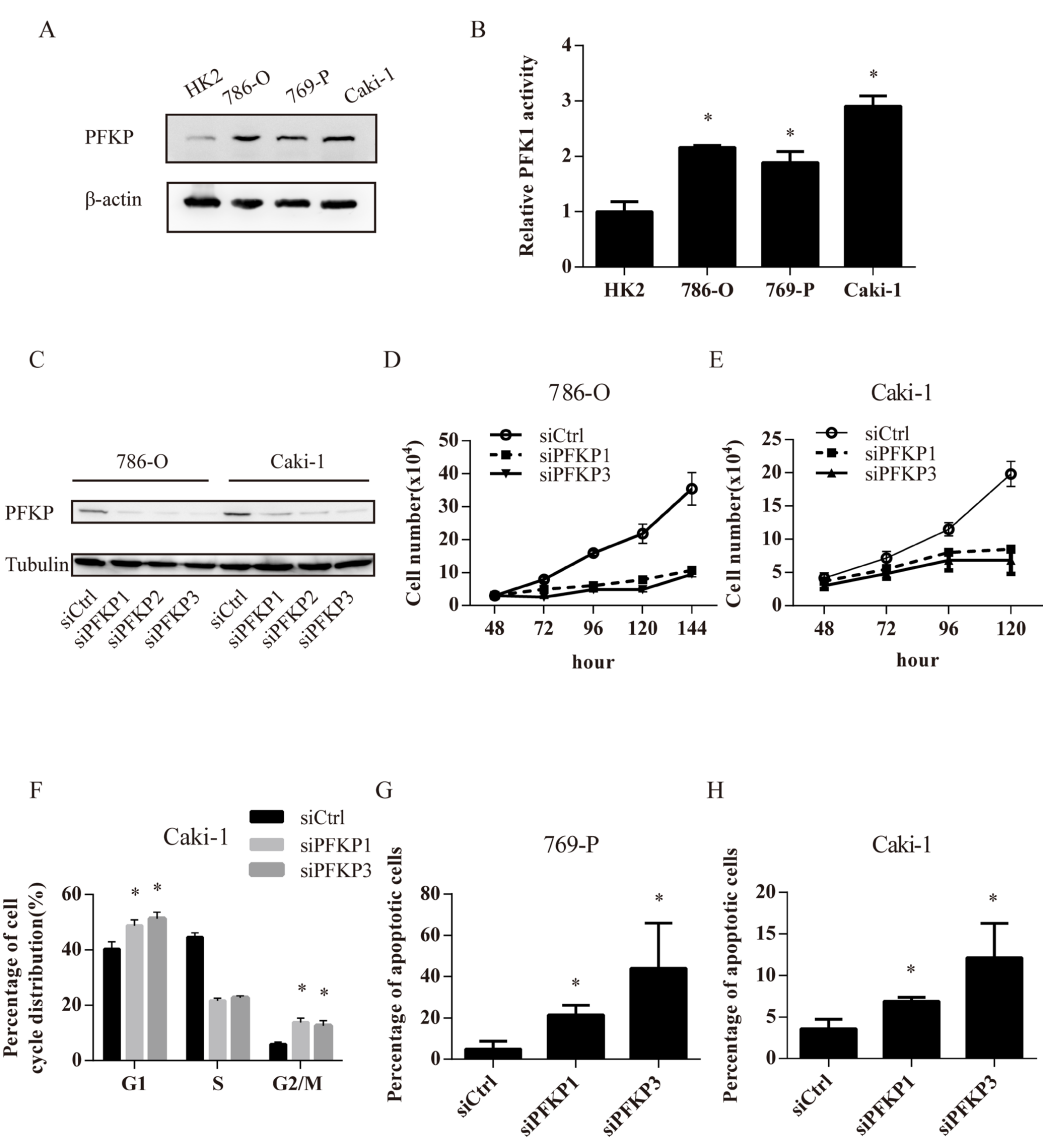
PFKP knockdown leads to impaired cell proliferation, cell cycle arrest and apoptosis in kidney cancer cell lines **A.** Western blot for protein extracts of normal kidney epithelial cell line HK-2 and 3 kidney cancer cell lines including 786-O, 769-P, Caki-1. **B.** PFK enzymatic activity in HK-2,786-O, 769-P and Caki-1 cell extracts. **C.** Western blot for protein extracts of 786-O and Caki-1 cells transiently transfected with control (siCtrl) or PFKP siRNAs (siPFKP1, siPFKP2 and siPFKP3) at 72 hours after transfection. **D.**-**E.** Cell growth curves of 786-O (D) and Caki-1 (E) cells transiently transfected with siCtrl, siPFKP1 or siPFKP3. **F.** Cell cycle analysis for Caki-1 cells transiently transfected with siCtrl, siPFKP1 or siPFKP3 at 72 hours after transfection based on Brdu incorporation assay. **G.**-**H.** Percentage of apoptotic cells in 769-P (G) and Caki-1 (H) cells transiently transfected with siCtrl, siPFKP1 or siPFKP3 at 72 hours after transfection according to annexin V and propidium iodide (PI) staining. * indicates *p* < 0.05 in B, F, G and H.

The level of PFKP protein was higher in three ccRCC cell lines including Caki-1, 786-O and 769-P than in a normal kidney cell line HK2 (Figure 2A). The activity of PFK1, the key enzyme in glycolysis, was increased in 786-O, 769-P and Caki-1 cells compared with HK2 cells, suggesting higher glycolytic activity in kidney cancer cells (Figure 2B).

To investigate the effect of PFKP suppression on kidney cancer cells, RNA interference (RNAi) was used to reduce PFKP levels in human kidney cancer cell lines. Transient transfections of 786-O and Caki-1 cells with three independent PFKP siRNA (siPFKP1, siPFKP2 and siPFKP3) all decreased the level of PFKP when compared to the non-target control group (siCtrl) (Figure 2C). We next assessed the effect of PFKP knockdown on kidney cancer cell proliferation. Cell numbers of 786-O and Caki-1 cells transiently transfected with siCtrl, siPFKP1 and siPFKP3 were counted at days 2, 3, 4, 5 and 6 after being plated at equal numbers. The capacity of proliferation was severely impaired in siPFKP1 or siPFKP3-transfected cells when compared to siCtrl-transfected cells (Figure 2D and 2E).

We next performed cell cycle analysis for siCtrl, siPFKP1 or siPFKP3-transfected cells using the BrdU incorporation assay. Reduced S-phase percentage was observed in siPFKP1 or siPFKP3-transfected Caki-1 cells, accompanied by both G1 and G2/M-phase arrest, when compared to siCtrl-transfected cells (Figure 2F). Moreover, PFKP knockdown induced apoptosis in 769-P and Caki-1 cells (Figure 2G and 2H). Therefore, both cell cycle alterations and apoptosis may account for decreased cell proliferation in siPFKP1 or siPFKP3-transfected cells.

Since PFKP was required for cell proliferation in kidney cancer cell lines, we next investigated whether PFKP was sufficient for promoting cell proliferation in low PFKP-expressing HK-2 cells. We generated HK-2 cells stably expressing either GFP or PFKP cDNA and found that ectopic PFKP expression did not increase cell proliferation (Supplementary Figure 1A and 1B). Therefore, PFKP is required but not sufficient for promoting cell proliferation.

In order to determine whether decreased cell proliferation and increased apoptosis was specific to PFKP depletion, we used three independent PFKL siRNA (siPFKL1, siPFKL2 and siPFKL3) and three independent PFKM siRNA (siPFKM1, siPFKM2 and siPFKM3) to suppress the expression of PFKL and PFKM (Supplementary Figure 2A-2D). Transient transfections of Caki-1 cells with siRNAs for PFKL or PFKM failed to alter cell proliferation or apoptosis when compared to the corresponding controls (Supplementary Figure 2E and 2F).

### Suppression of PFKP leads to alterations in glycolysis, TCA cycle and pentose phosphate pathway (PPP) in kidney cancer cells

**Figure 3:**
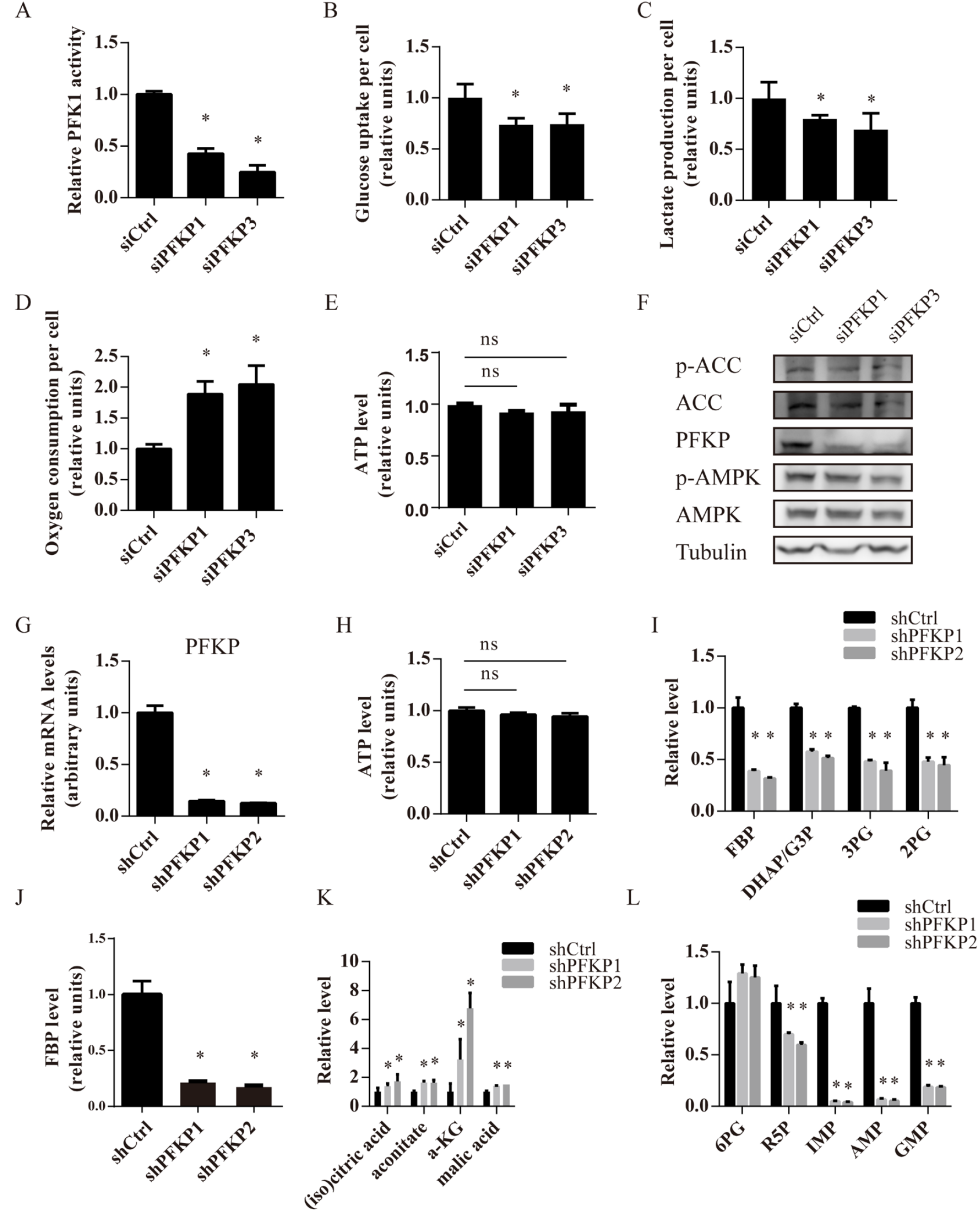
Suppression of PFKP inhibits glycolysis, promotes oxygen consumption and increases nucleotide biosynthesis in kidney cancer cells **A.** PFK enzymatic activity of Caki-1 cells transiently transfected with siCtrl, siPFKP1 or siPFKP3. **B.**-**E.** Glucose uptake (B), lactate production (C), oxygen consumption rates (D) and ATP levels (E) per cell in Caki-1 cells transiently transfected with siCtrl, siPFKP1 or siPFKP3 at 72 hours after transfection. **F.** Western blot analysis for Caki-1 cells transiently transfected with siCtrl, siPFKP1 or siPFKP3 using antibodies to p-ACC, ACC, PFKP, p-AMPK, AMPK and tubulin at 72 hours after transfection. **G.** Real time PCR analysis for Caki-1 cells stably transfected with shRNAs for control (shCtrl) or PFKP (shPFKP1 and shPFKP2). **H.** ATP levels per cell in Caki-1 cells stably transfected with shCtrl, shPFKP1 or shPFKP2. **I.** Measurement of glycolytic metabolites in Caki-1 cells stably transfected with shCtrl, shPFKP1 or shPFKP2 using LC-MS. **J.** Measurement of FBP in Caki-1 cells stably transfected with shCtrl, shPFKP1 or shPFKP2 using an enzymatic method **K.**-**L.** Measurement of metabolites in TCA cycle (K) and *de novo* nucleotide biosynthesis (L) in Caki-1 cells stably transfected with shCtrl, shPFKP1 or shPFKP2 using LC-MS. * indicates *p* < 0.05 in A-D, G and I-L. The symbol of ns indicates no significant difference in E and H. The following abbreviations are used, fructose 1,6 phosphate (FBP), glyceraldehyde 3-phosphate (G3P), dihydroxy-acetone-phosphate (DHAP), 2-phosphoglyceric acid (2PG), 3-phosphoglyceric acid (3PG), α-ketoglutaric acid (α-KG), ribose 5-phosphate (R5P), 6-phospho-D-gluconate (6PG).

PFKP knockdown significantly decreased total PFK1 enzyme activity (Figure 3A). Given that PFK1 was one of key enzymes in glycolysis, we compared the glycolytic activity of Caki-1, 786-O and 769-P cells transfected with either control or PFKP siRNAs by measuring glucose uptake and lactate production per cell 48 hours after transfection. As expected, PFKP knockdown reduced glucose uptake and lactate production per cell in all three kidney cancer cell lines, indicating declined aerobic glycolysis (Figure 3B and 3C, Supplementary Figure 3A, 3B, 3D and 3E). Since GLUT1 was the major glucose transporter in renal cancer cells [23], we investigated whether PFKP knockdown decreased GLUT1 expression. We found that PFKP knockdown did not alter the level of GLUT1 (Supplementary Figure 4). In addition, we observed increased oxygen consumption rates in Caki-1, 786-O and 769-P cells transfected with siPFKP1 or siPFKP3 when compared to siCtrl-transfected cells, indicating that PFKP knockdown promoted mitochondrial respiration (Figure 3D, Supplementary Figure 3C and 3F). We next explored whether PFKP knockdown altered cellular energy status by examining cellular ATP levels and the activity of AMPK, a cellular energy sensor. Cellular ATP levels were similar in kidney cancer cells transfected with control or PFKP siRNAs (Figure 3E). Levels of phosphorylated AMPK and its substrate, acetyl-CoA carboxylase (ACC), were hardly altered by PFKP suppression in Caki-1 cells (Figure 3F). Our results suggest that PFKP suppression decreased glycolytic activity and induced oxygen consumption without affecting cellular energy status in kidney cancer cells.

In order to further examine the effects of PFKP suppression on glucose metabolism, we established Caki-1 cells stably transfected with a non-target control shRNA (shCtrl) or two independent shRNAs for PFKP (shPFKP1 and shPFKP2). Stable transfection of Caki-1 cells with shPFKP1 and shPFKP2 led to a more than 80% decline in PFKP mRNA levels compared to the control (Figure 3G). Similar to what was observed in PFKP siRNA-mediated transient transfection, shRNA-mediated suppression of PFKP decreased glycolytic activity, induced oxygen consumption and did not alter cellular ATP levels (Figure 3H, Supplementary Figure 5A-5C). Liquid chromatography mass spectrometry (LC-MS) results showed that PFKP knockdown led to decreased levels of fructose 1,6-bisphosphate (FBP), the product of PFKP, and its downstream glycolytic metabolites such as glyceraldehyde 3-phosphate (G3P), dihydroxy-acetone-phosphate (DHAP), 3-phosphoglyceric acid (3PG) and 2-phosphoglyceric acid (2PG) (Figure 3I). We further confirmed that levels of fructose 1,6-bisphosphate were reduced in Caki-1 cells stably transfected with shPFKP1 and shPFKP2 using an enzymatic assay (Figure 3J). In addition, we observed an increase in abundance of metabolites in the TCA cycle including citric acid/isocitric acid, aconitate, α-ketoglutaric acid and malic acid in Caki-1 cells stably transfected with shPFKP1 and shPFKP2, indicating increased TCA cycle flux, which was consistent with our finding of increased oxygen consumption rates (Figure 3K).

Interestingly, we found reduced abundance of metabolites in *de novo* nucleotide biosynthesis, including ribose 5-phosphate (R5P), IMP, AMP and GMP (Figure 3L). R5P can be synthesized from both the oxidative and non-oxidative branch of PPP. In our study, the level of 6-phospho-D-gluconate (6PG) was not significantly changed in Caki-1 cells stably transfected with shPFKP1 or shPFKP2, suggesting that the oxidative branch of the PPP was not affected by PFKP knockdown (Figure 3L). Therefore, it is possible that, less G3P and DHAP due to PFKP suppression, prevented non-oxidative PPP intermediates from generating R5P, leading to reduced *de novo* nucleotides biosynthesis.

### PFKP suppression activates p53 in kidney cancer cells

**Figure 4:**
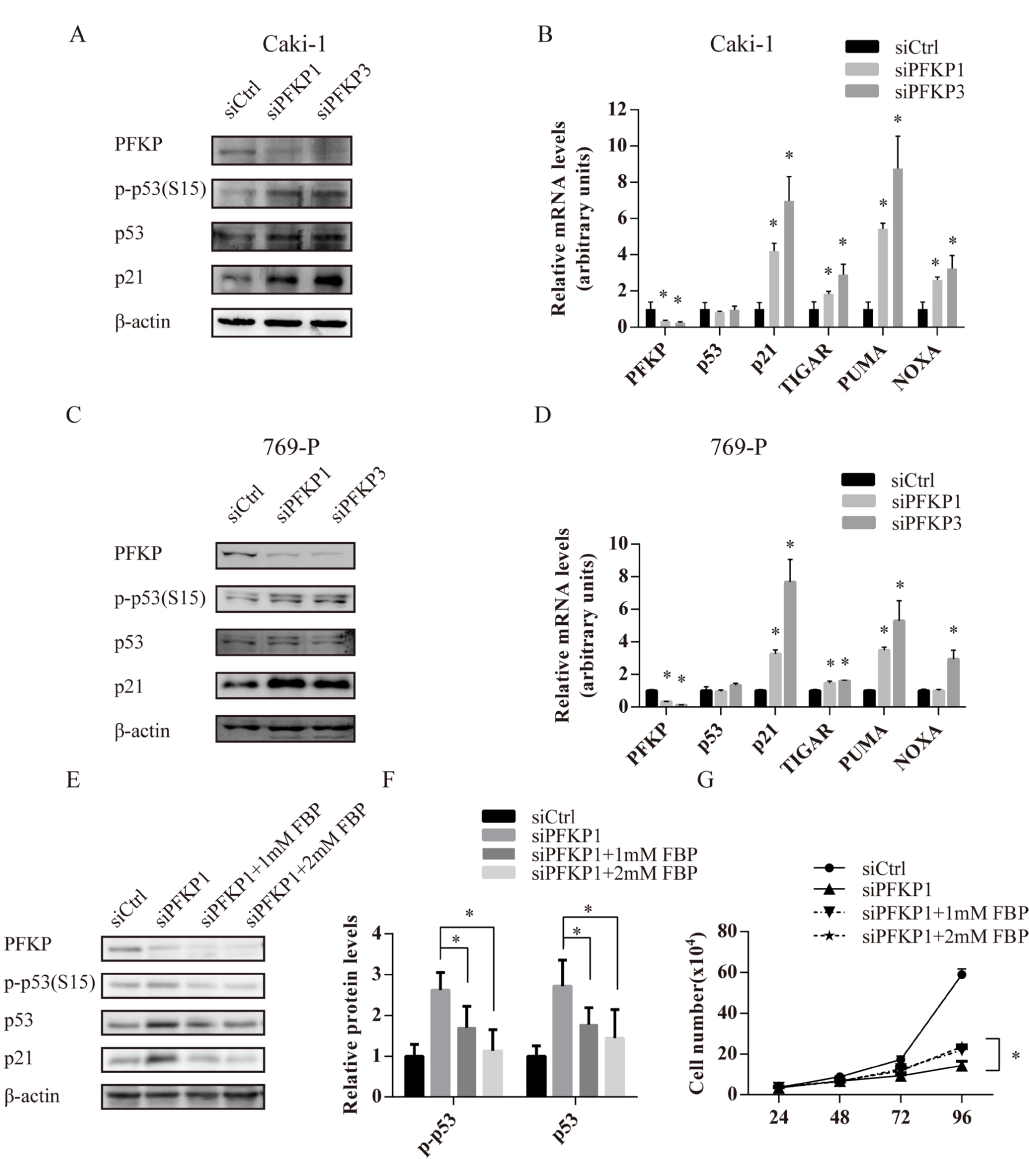
FBP deficiency due to PFKP suppression activates the p53 pathway in kidney cancer cells **A.**, **C.** Western blot analysis for Caki-1 (A) and 769-P (C) cells transiently transfected with siCtrl, siPFKP1 or siPFKP3 using antibody to PFKP, p-p53, p53, p21and actin at 72 hours after transfection. **B.**, **D.** Real time PCR analysis for Caki-1 (B) and 769-P (D) cells transiently transfected with siCtrl, siPFKP1 or siPFKP3 at 72 hours after transfection. **E.** Western Blot analysis for Caki-1cells transiently transfected with siCtrl, siPFKP1 or siPFKP3 after 1mM or 2mM FBP treatment at 72 hours after transfection. **F.** Quantification of western blot for p-p53, p53 normalized by corresponding actin levels using Gel-Pro analyzer 4. **G.** Cell growth curves of Caki-1cells transiently transfected with siCtrl, siPFKP1 or siPFKP3 after 1mM or 2mM FBP treatment. * indicates *p* < 0.05 in B and D.

Cell cycle alterations and apoptosis observed in siPFKP1 or siPFKP3-transfected cells led us to examine the activity of p53, a master regulator for cell cycle and apoptosis in response to various stress [24–27]. Caki-1and 769-P cells transfected with siPFKP1 or siPFKP3 displayed higher levels of p53 phosphorylated on Ser-15 and p21 when compared to siCtrl-transfected cells (Figure 4A and 4C). Moreover, mRNA levels of p53 targets genes including p21, PUMA, NOXA and TIGAR were increased when PFKP was suppressed in Caki-1and 769-P cells (Figure 4B and 4D). Up-regulation of the cell cycle inhibitor p21 and pro-apoptotic PUMA and NOXA correlated with cell cycle arrest and apoptosis phenotypes resulted from PFKP knockdown.

Given the deficiency of FBP, the product of PFKP, and its downstream glycolytic metabolites due to PFKP suppression, we speculated that FBP reduction might serve as the metabolic stress signal to activate p53, the cellular sensor for various stress. Adding FBP in the culture medium was reported to protect primary cultures of astrocytes from hypoxic damage [28]. We treated Caki-1 cells transfected with siPFKP1 with 1 mM FBP and found that intracellular FBP level was restored (Supplementary Figure 6A). Although FBP treatment did not alter the status of phosphorylated p53 and p21 in parental Caki-1 cells (Supplementary Figure 6B), treating Caki-1 cells transfected with siPFKP1 with 1 mM or 2 mM FBP reduced p53 activation (Figure 4E–F). Moreover, FBP treatment of Caki-1 cells transfected with siPFKP1 partly restored cell proliferation (Figure 4G) without reducing the number of apoptotic cells (Supplementary Figure 6C).

### Activated p53 contributes to impaired cell proliferation and metabolic alterations induced by PFKP knockdown in kidney cancer cells

**Figure 5:**
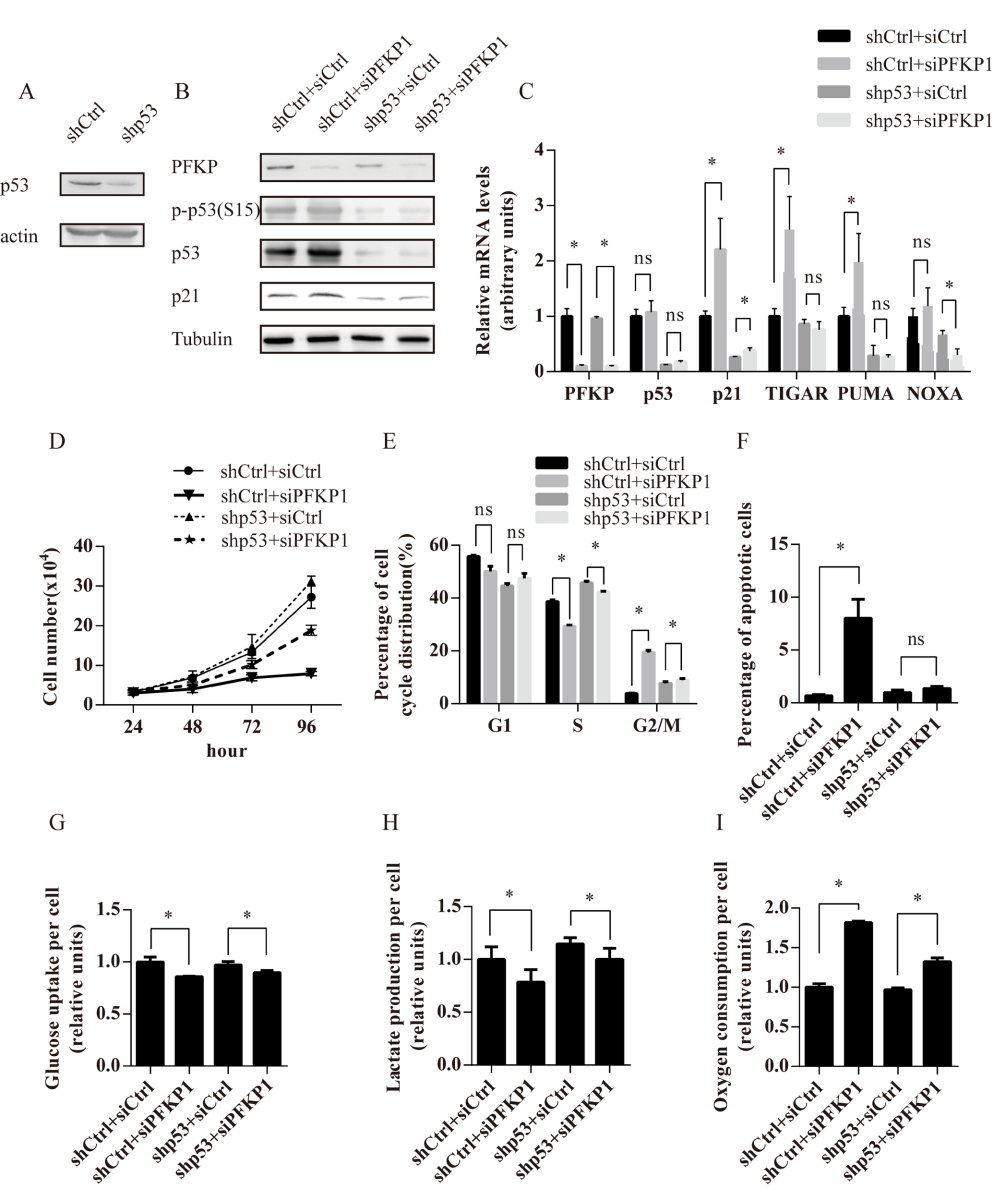
The p53 pathway mediates cell proliferation and metabolic defects induced by PFKP suppression in kidney cancer cells **A.** Western blot analysis for Caki-1 cells stably transfected with shRNAs for control (shCtrl) or p53 (shp53). **B.**-**I.** Caki-1 cells stably transfected with shCtrl or shp53 were transiently transfected with siCtrl or siPFKP1 and subjected to various analysis including Western blot (B), real time PCR (C), Brdu incorporation (D), annexin V and PI staining (F), glucose uptake (G), lactate production (H) and oxygen consumption rate (I) at 72 hours after transfection. * indicates *p* < 0.05 in C, E-I.

To test if p53 was required for impaired cell proliferation and metabolic alterations in PFKP knockdown cells, we established Caki-1 cells stably transfected with control (shCtrl) or p53 shRNA (shp53). Stable transfection of p53 shRNA effectively reduced the protein level of p53 (Figure 5A). Transient transfection of siPFKP1 led to reduction in PFKP protein levels in both shCtrl and shp53-transfected Caki-1 cells (Figure 5B). The up-regulation of p53 phosphorylated on Ser-15 and p21 after siPFKP1transfection was blocked by p53 knockdown (Figure 5B). Moreover, the up-regulation of p21, PUMA and TIGAR mRNA in siPFKP1-transfected cells was partially or completely blocked by suppression of p53 (Figure 5C).

Growth kinetics of Caki-1 cells with different levels of p53 and PFKP were compared by counting cell numbers at days 1, 2, 3 and 4 after transfection and plating equal number of cells. Caki-1 cells stably transfected with shCtrl and shp53 showed a reduction in cell proliferation when transfected with siPFKP1 compared to siCtrl-transfected cells (Figure 5D). However, the degree of impairment in cell proliferation was less in Caki-1 cells stably transfected with shp53 as compared to shCtrl cells (Figure 5D). Transient siPFKP1 transfection led to cell cycle alterations including reduced S-phase percentage, G1 and G2/M-phase arrest (Figure 2F). Similarly, PFKP knockdown in Caki-1 cells stably transfected with shCtrl showed a reduction in S-phase population accompanied by G2/M-phase arrest (Figure 5E). Reduction in S-phase population and G2/M-phase arrest was alleviated in Caki-1 cells stably transfected with shp53 as compared to shCtrl-transfected cells (Figure 5E). Moreover, suppression of p53 completely blocked apoptosis induced by PFKP knockdown in Caki-1 cells (Figure 5F). Therefore, p53 plays an important role in mediating cell cycle alterations and apoptosis resulted from PFKP knockdown in kidney cancer cells.

In order to examine whether p53 contributes to metabolic defects resulting from suppression of PFKP, Caki-1 cells stably transfected with shCtrl and shp53 were transiently transfected with siCtrl and siPFKP1 and assayed for metabolic activities. Suppression of PFKP led to a more modest inhibition of lactate production as well as a milder increase in oxygen consumption rate in Caki-1 cells stably transfected with shp53 when compared to shCtrl-transfected cells (Figure 5H–5I). However, the change in glucose uptake was not affected by p53 depletion (Figure 5G). Taken together, our findings suggest that p53 contributes to impaired cell proliferation and metabolic alterations in PFKP knockdown cells.

### PFKP suppression inhibits tumor growth *in vivo*

**Figure 6:**
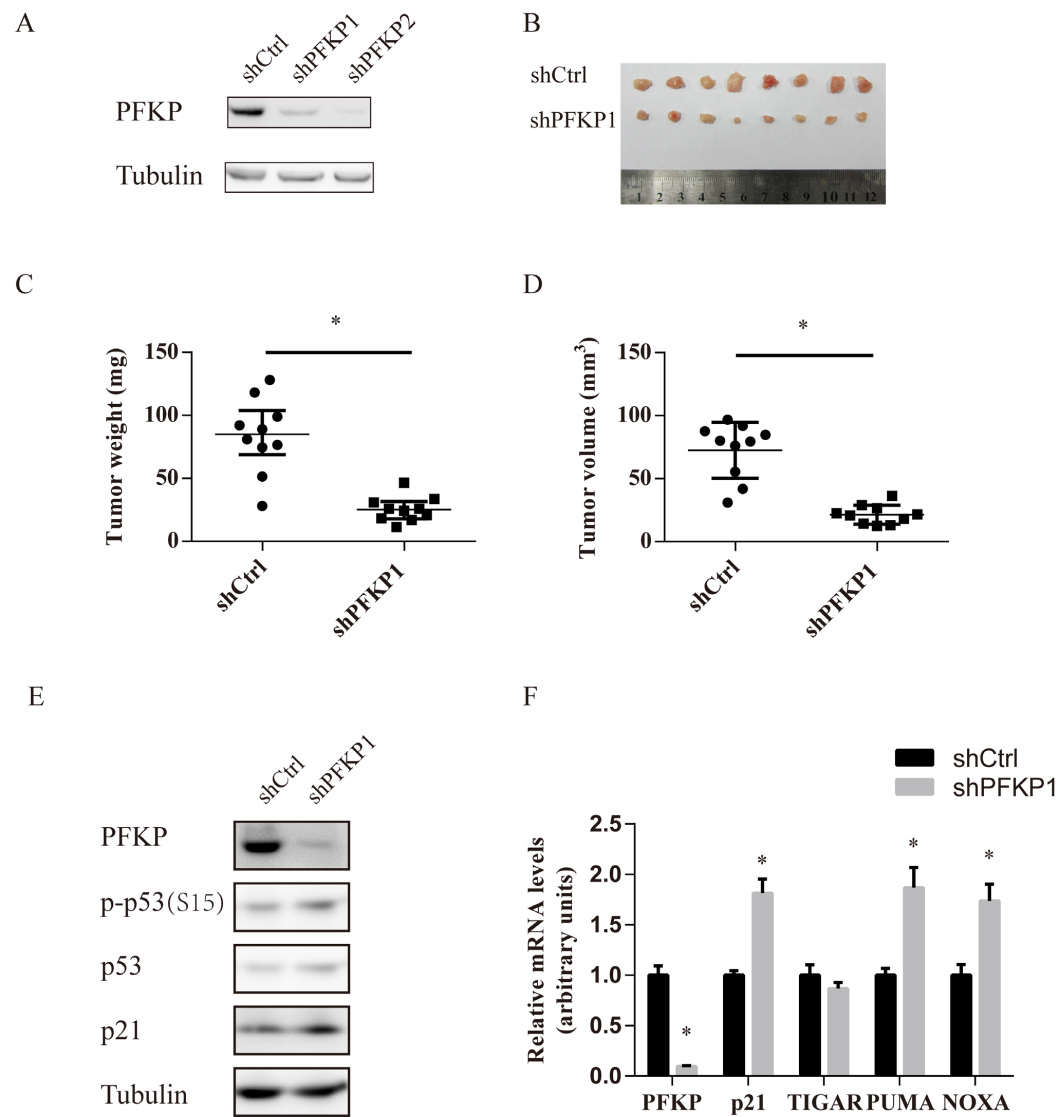
PFKP suppression inhibits renal xenograft tumor growth **A.** Western blot analysis for Caki-1 cells stably transfected with shCtrl, shPFKP1 or shPFKP2 using antibody to PFKP and tubulin. **B.** Picture of subcutaneous tumors from nude mice subcutaneously injected with Caki-1 cells stably transfected with shCtrl or shPFKP1. **C.**-**D.** Weight (C) and size (D) of subcutaneous tumors from nude mice subcutaneously injected with Caki-1 cells stably transfected with shCtrl or shPFKP1. (*n* = 10). **E.**-**F.** Western blot (E) and real time PCR analysis (F) for protein and RNA extracts of subcutaneous tumors from nude mice subcutaneously injected with Caki-1 cells stably transfected with shCtrl or shPFKP1.

To examine the effects of PFKP knockdown on the *in vivo* tumorigenicity of kidney cancer cells, Caki-1 cells stably transfected with shCtrl or two independent shRNAs for PFKP (shPFKP1 and shPFKP2) were injected subcutaneouly into nude mice and examined for tumor formation. 20 nude mice were divided into two groups of 10 mice each. Each group of 10 mice were injected with a control inoculation in one flank and a shPFKP1 or shPFKP2-transfected inoculation in the other. Tumor growth was significantly reduced in the shPFKP1 or shPFKP2 group compared with the shCtrl group (Figure 6B–6D, Supplementary Figure 7A-7C). Moreover, western blot and quantitative PCR analyses of protein and RNA expression for the excised tumors confirmed maintenance of both the PFKP knockdown and increased p53 activity phenotypes (Figure 6E and 6F). Our findings show that PFKP suppression inhibits ccRCC tumor growth *in vivo*.

## DISCUSSION

In the present study, we reported for the first time that PFKP was the predominant isoform of PFK in human ccRCC patient tissues. We found that PFKP was overexpressed in 19 ccRCC tissue specimens and 3 ccRCC cell lines compared to normal tissues and cells. Knockdown of PFKP, but not PFKL or PFKM, inhibited kidney cancer cell growth and induced apoptosis and cell cycle arrest. Decreased PFKP expression inhibited aerobic glycolysis, PPP and nucleotide biosynthesis while increased TCA cycle activity in kidney cancer cells. Moreover, FBP depletion followed by PFKP suppression activated p53 pathway, which played an important role in mediating the proliferative and metabolic alterations in response to PFKP knockdown. Therefore, our data have revealed a novel role of PFKP in regulating kidney cancer cell metabolism and proliferation and uncovered the underlying mechanism.

Our study and previous findings report that PFKP catalyzing the formation of fructose 1,6-bisphosphate from fructose 6-phosphate is up-regulated in ccRCC. FBP1 which hydrolyzes fructose 1,6-bisphosphate to fructose 6-phosphate has been reported to be down-regulated in ccRCC [11]. Both FBP1 depletion and PFKP up-regulation ensure increased glycolytic flux to support macromolecular biosynthesis for cell proliferation. In fact, the role of PFKP in other cancer types such as breast and liver cancer has been reported. Krüppel-like factor 4 (*KLF4*) overexpression increased PFKP expression as well as glycolytic activity in breast cancer cell lines [34]. In hepatocellular carcinoma, PFKP was reported to be regulated by Tat-activating regulatory DNA-binding protein through microRNA 520 [35]. Importantly, recent determination of mammalian PFKP crystal structure can provide us with tremendous help in therapeutic targeting the enzyme to control aerobic glycolysis in different cancers[36].

Intriguingly, we found that depletion of PFK1 suppressed levels of metabolites in *de novo* nucleotide synthesis pathway, including R5P, IMP, AMP and GMP. We did not observe a significant change in 6PG in Caki-1 cells stably transfected with shPFKP1 or shPFKP2, indicating that the oxidative branch of the PPP might not be responsible for reduced FBP. Indeed, it has been reported that cancer cells tend to generate R5P through the non-oxidative PPP [37]. Several enzymes in non-oxidative PPP are overexpressed in various tumor tissues including ccRCC [38–40]. One possible mechanism is that decreased G3P and DHAP due to PFKP suppression prevents the non-oxidative branch of PPP from generating R5P.

We explored the mechanism by which depletion of PFKP activated p53 in ccRCC cells in many ways. Apart from genotoxic stress, p53 integrates signals induced by various stress, such as energy depletion, ROS, oncogene activation, ribosomal stress, hypoxia, telomere erosion, nutrient deprivation [24]. Lack of alterations in ATP levels and AMPK phosphorylation status in PFKP knockdown cells suggest that p53 activation was not due to energy depletion. We found that PFKP depletion did not shift the GSH/GSSG redox balance or ROS levels (data not shown). Although we observed decreased nucleotide biosynthesis in PFKP stably knockdown cells compared to control cells, supplementation of dNTPs did not rescue cell proliferation and p53 activation phenotypes induce by PFKP suppression (data not shown). Intriguingly, treating Caki-1 cells transfected with siPFKP1 with FBP reduced p53 activation and partly restored cell proliferation (Figure 4E and 4F). Metabolic changes resulted from FBP deficiency might serve as a metabolic stress to activate the p53 pathway. One possible explanation is that guanosine 5′-monophosphate synthase (GMPS) might sense the depletion of GMP and activate p53. It has been reported that in response to GMP deprivation, GMPS promotes p53 stabilization by preventing p53 ubiquitination [41]. Another possible explanation is that FBP deficiency results in decreased flux into serine synthesis pathway which has been reported to activate p53 [25, 42–45]. We have found that serine hydromethyltransferase 2 (SHMT2) in the serine and glycine sythesis pathway is up-regulated in ccRCC (our unpublished data), indicating increased demand for serine or glycine in ccRCC. The detailed mechanism by which FBP regulates p53 pathway remains be elucidated.

We have found that the mRNA of TIGAR is up-regulated in response to transient PFKP siRNA transfection in ccRCC cell lines (Figure 4B and 4D). However, the expression of TIGAR is not increased in Caki-1 cells stably transfected with shPFKP1 or shCtrl in the xenograft model (Figure 6F). In addition to the difference between cultured cancer cells and *in vivo* tumor environment, the reported adaptation in TIGAR expression from acute to chronic conditions could contribute to the lack of TIGAR induction in tumors of Caki-1 cells stably transfected with shPFKP1 [46, 47].

## MATERIALS AND METHODS

### Samples

Tissue samples were obtained from the Shanghai First People's Hospital, Shanghai Jiao Tong University School of Medicine. Investigation conducted in accordance with ethical standards has been approved by the authors’ institutional review board. Details of enrolled subjects are summarized in Supplementary Table 1.

### Cell culture

Three ccRCC cell lines (786-O, 769-P, Caki-1) were used [29–31]. The 786-O and 769-P cell lines were obtained from the Type Culture Collection of the Chinese Academy of Sciences, Shanghai, China. The HK-2 and Caki-1 cell lines were gifts from Dr. Benkang Shi at Shandong University, China. All cells were cultured in RPMI 1640 (Invitrogen Life Technologies, USA) supplemented with 10% FBS, 2 mmol/l L-glutamine, 100 unit /ml penicillin, and 100 g/ml streptomycin at 37°C in humidified 5% CO_2_ atmosphere.

### Immunohistochemistry

Sections were deparaffinized and rehydrated followed by antigen retrieval. Next, sections were treated with 3% hydrogen peroxide in methanol to block endogenous peroxidase activity. After a 30 minute FBS treatment to block non-specific protein binding, the primary anti-PFKP antibody (Cell Signaling Technology, USA) was applied followed by HRP-conjugated secondary antibody incubation (Santa Cruz Biotechnology, USA). Apply DAB substrate solution was applied before hematoxylin counterstaining. Negative control assays were performed using normal IgG. All images were photographed with a ×20 objective lens using the Nikon Eclipse Ti microscope.

### siRNA transfection

Custom-designed siRNAs directed against PFKP (siPFKP1 GGUGUUCCUUCCAGAAUCUTT, siPFKP2 GGCUGAAGGAGCAAUUGAUTT, siPFKP3 GCUCCAUUCUUGGGACAAATT), PFKM (siPFKM1 CUGCAUCGGAUCAUGGAAATT, siPFKM2 GCUUCUAGCUCAUGUCAGATT, siPFKM3 GCUUGGUGUUAAGGAAUGATT), PFKL (siPFKL1 GCAUUUAUGUGGGUGCCAATT, siPFKL2 CGGAAGGUAAGAUCUCAGATT, siPFKL3 GACACACAGUAUACGUGGUTT) were synthesized and annealed (GenePharma, China). Using lipofectamine RNAiMAX (Invitrogen Life Technologies, USA), cells were transfected with siRNAs according to the manufacturer's instructions.

### ShRNA and cDNA transfection

The PFKP (TRCN0000037775, TRCN0000199816) and p53 (TRCN0000003753) shRNA construct were obtained from TRC Lentiviral shRNA Libraries (Broad Institute, USA). The cDNA encoding human PFKP was obtained by reverse transcription PCR and verified by sequencing. The PFKP cDNA was then subcloned into the lentiviral vector pLVX-IRES-ZsGreen1(Clontech). The human PFKM and PFKL cDNAs were purchased from Open Biosystems (GE Healthcare, USA). The PFKP, p53 shRNA and PFKP cDNA were transfected into 293T cells with the packaging plasmids, PMD2G and PSPAX2, using Lipofectamine 2000 (Invitrogen Life Technologies, USA) according to the manufacturer's instructions. Viral supernatant was collected after 48 hours. 5 ml viral supernatant and 5 ml of fresh medium were added to HK-2 and Caki-1 cells plated in 10 cm dishes. 24 hours later, the medium was replaced with fresh medium. HK-2 cells stably transfected with PFKP cDNA were selected using flow cytometry. Caki-1 cells stably transfected with different shRNAs were selected using 1 μg/ml puromycin.

### RNA extraction, cDNA synthesis and real-time PCR

Total RNA was extracted using TRIzol (Invitrogen Life Technologies, USA) according to the manufacturer's instructions. Total RNA was reverse transcribed into cDNA using the PrimeScript ™ RT reagent kit (Takara Bio Inc., Japan). The SYBR® green Premix Ex Taq TM kit (Takara Bio Inc., Japan) was used for real-time PCR analysis by a StepOnePlus™ Real-Time PCR System (Applied Biosystems, USA). Absolute quantification of mRNA abundance was performed according to user's guide of Applied Biosystems. Diluted linear plasmids that contain the human PFKP, PFKL or PFKM cDNA were used as standards, respectively. All primers are listed in Supplementary Table 2.

### Western blot analysis

Total protein extracts were obtained using the RIPA lysis buffer (Sigma, USA). Protein concentration was measured using a BCA Protein Assay kit (Pierce, USA). Protein samples were resolved in 7.5 and 15% SDS-PAGE gels and transferred to Immuno-blot PVDF Membrane (Bio-Rad, USA). The membranes were blocked using 5% non-fat milk in Tris-buffered saline with Tween-20. The membranes were then incubated with primary antibodies followed by incubation with secondary peroxidase labeled anti-rabbit or anti-mouse antibodies (Santa Cruz Biotechnology, USA). The protein signals were detected using an enhanced chemiluminescent solution (Millipore, USA). Primary antibodies used include anti-tubulin, anti-actin (Sigma, USA), anti-PFKP, anti-p53, anti-phospho-p53, anti-p21, anti-ACC, anti-phospho-ACC, anti-AMPK, anti-phospho-AMPK (Cell Signaling Technology, USA), anti-PFKM, anti-PFKL, anti-GLUT1 and anti-V5 (Proteintech Group, USA).

### Cell proliferation

24h hours after siRNA transfection, cells were replated in 6-well plates with a density of 4000 cells/well. Cell numbers were counted at day 2, 3, 4, 5 after replating. Alternatively, cells were reversely transfected with siRNAs and plated in 6-well plates with a density of 4000 cells/well at day 1. Cell numbers were counted at day 2, 3, 4, 5 after plating.

### Metabolic assays

To measure oxygen consumption, 80% confluent cells in 10 cm dishes were trypsinized and resuspended in 2 ml fresh medium. Oxygen consumption was measured using an Oxytherm system (Hansatech, UK). To measure glucose uptake and lactate production, 48 hours after cells were plated in 6-well plates, culture medium was collected and investigated using the glucose assay kit (Shanghai Rongsheng Biotech, China) and lactate assay kit (Sigma, USA) according to manufacturers’ instructions.

Enzymatic measurements of FBP was conducted according to the described procedure with slight modifications [32]. Briefly, the neutralized extract was added in the reaction buffer containing 0.5 mM Na-EDTA, 50 mM Tris-HCl pH 7.5 and 0.2 mM NADH. Addition of 0.2U glycerol-3-phosphate dehydrogenase (Sigma, USA) and 1U triosephosphate isomerase (Sigma, USA) started the reaction and depleted endogenous DHAP and GA3P. After the reaction ended, 0.2U aldolase (Sigma, USA) was added to measure FBP. Absorbance was recorded at 340 nm at room temperature using the Thermo Scientific Multiskan GO spectrophotometer.

PFK1 activity was measured according to the described procedure with slight modifications [33]. Briefly, 2 μg cell lysate was added to 100 ul of reaction buffer containing 50 mM Tris-HCl pH 7.5, 100 mM KCl, 5 mM MgCl_2_, 1 mM ATP, 0.2 mM NADH, 5 mM Na_2_HPO_4_, 0.1 mM AMP, 1 mM NH_4_Cl, 5 mM fructose 6-phosphate, 0.5 U of triose phosphate isomerase (Sigma, USA), 0.1 U of aldolase (Sigma, USA) and 0.1 U of α-glycerophosphate dehydrogenase (Sigma, USA). Absorbance was recorded at 340 nm at room temperature every 120 s for 28 min using the Thermo Scientific Multiskan GO spectrophotometer.

### Metabolomic quantitation by high-performance liquid chromatography–mass spectrometry (HPLC-MS)

For metabolite extraction, cells were harvested and re-suspended in a solution of acetonitrile, methanol and water (2/2/1 by v/v; all HPLC grade) supplemented with internal standards. Following centrifugation at 12000 × *g* for 10 min at 4°C, supernatants were dried under N_2_ stream at room temperature. Resulting pellets were dissolved and the protein concentration determined with a Pierce BCA Protein Assay Kit (Thermo Scientific, USA) according to the manufacturer's instructions.

Liquid chromatography - tandem mass spectrometry data were acquired using a TSQ Vantage triple quadrupole mass spectrometer coupled to a Dionex Ultimate 3000 UHPLC system (Thermofisher Scientific, San Jose, CA, USA). Chromatographic separation was performed on a Acquity UPLC BEH Amide column (2.1 × 100 mm, 1.7 μm) with a VanGuard BEH Amide guard column (2.1 × 10 mm, 1.7 μm; Waters, Ireland) at a flow rate of 400 μL/min. The mobile phase was composed of buffer A (20 mM ammonium hydroxide, 20 mM ammonium acetate in 95% (vol/vol) water, 5% (vol/vol) acetonitrile (pH=9.0) and buffer B (100% acetonitrile). Gradient elution profile was 5% B (0.0-0.5 min), 5%-30% B (0.5-3.0 min), 30%-50% B (3.0-7.5 min), 50% B (7.5-9.0 min), followed by re-equilibration at 5% B for 6 min. The ion spray voltage was 3000 V in positive mode and 2500 V in negative mode. The vaporizer temperature and capillary temperature was 300°C and 320°C, respectively. The sheath gas and aux gas pressure were 40 arb and 10 arb, respectively. The raw data files were processed using LCquan 2.7 software (Thermofisher Scientific, USA) to generate chromatographic peak areas of each metabolite and their response ratios to internal standard. Then the response ratios were compared between different treatment groups.

### Statistical analysis

Statistical analysis was performed using Prism 5 (GraphPad Software, USA) and SPSS (IBM, USA). Experiments were performed at least three times independently. P values were 2-sided and p < 0.05 was considered statistically significant.

## SUPPLEMENTARY FIGURES AND TABLES


