# Supplementary material for: The platelet isoform of phosphofructokinase contributes to metabolic reprogramming and maintains cell proliferation in clear cell renal cell carcinoma
Source: Oncotarget. 2016 Mar 25;7(19):27142–57. doi: 10.18632/oncotarget.8382 (PMC5053638; doi:10.18632/oncotarget.8382)
Supplement: Supplementary file 1 [file oncotarget-07-27142-s001.pdf]

The platelet isoform of phosphofructokinase contributes to metabolic reprogramming and maintains cell proliferation in clear cell renal cell carcinoma

Supplementary Material

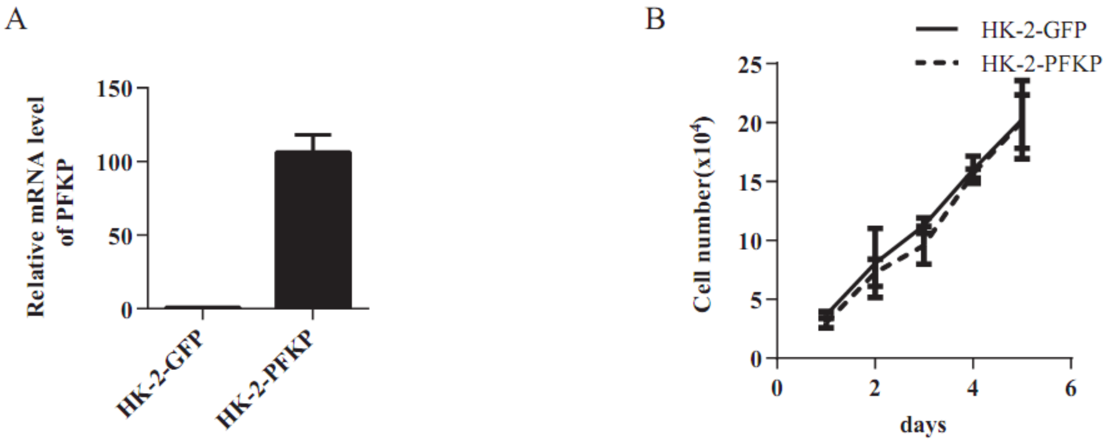

Supplementary Figure S1: Ectopic PFKP expression does not alter HK-2 cell proliferation.

(A) Real time PCR analysis for HK-2 cell stably transfected with GFP or PFKP cDNA.

(B) Cell growth curve for HK-2 cell stably transfected with GFP or PFKP cDNA.

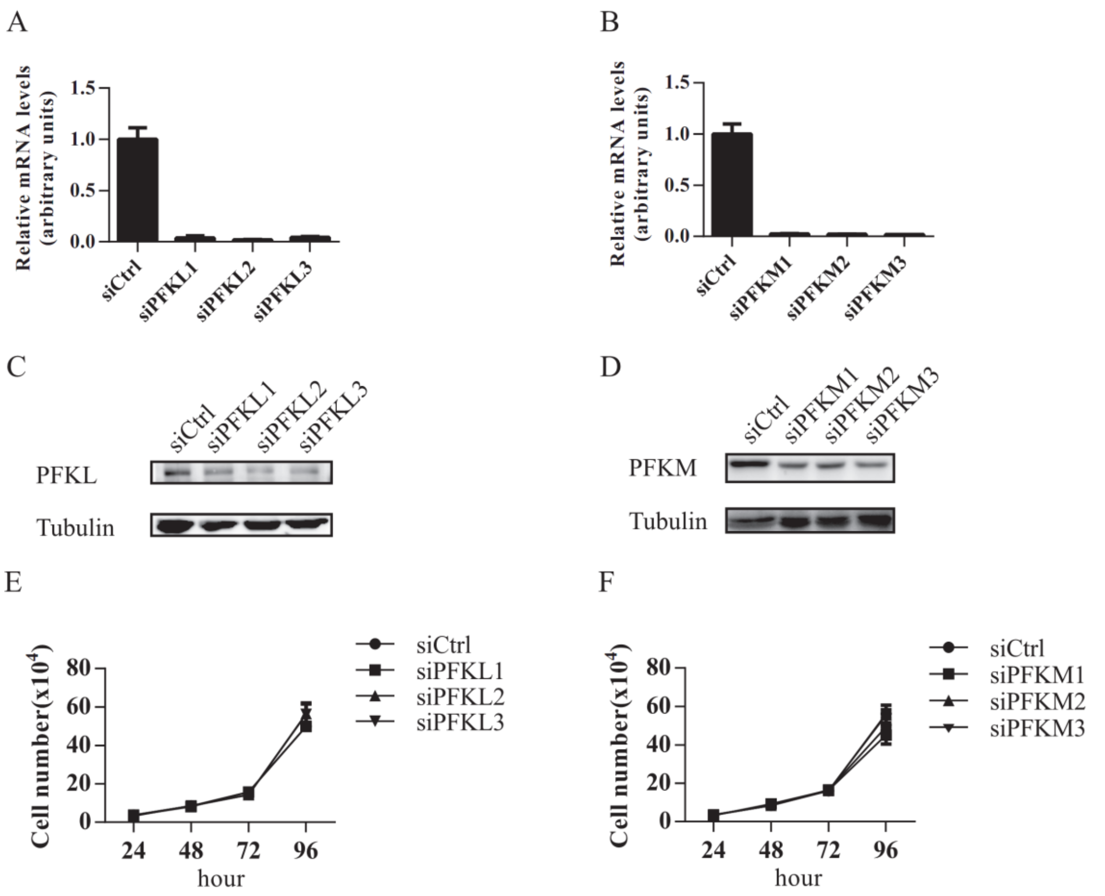

Supplementary Figure S2: PFKL and PFKM are not required for cell proliferation in kidney cancer cell lines.

(A-B) Real time PCR analysis for Caki-1 cells transiently transfected with control (siCtrl), PFKL siRNAs (siPFKL1, siPFKL2 and siPFKL3) (A) or PFKM siRNAs (siPFKM1, siPFKM2 and siPFKM3) (B) at 72 hours after transfection.

(C-D) Western blot for protein extracts of Caki-1 cells transiently transfected with control (siCtrl), PFKL siRNAs (siPFKL1, siPFKL2 and siPFKL3) (C) or PFKM siRNAs (siPFKM1, siPFKM2 and siPFKM3) (D) at 72 hours after transfection.

(E-F) Cell growth curves of Caki-1 cells transiently transfected with control (siCtrl), PFKL siRNAs (siPFKL1, siPFKL2 and siPFKL3) (E) or PFKM siRNAs (siPFKM1, siPFKM2 and siPFKM3) (F).

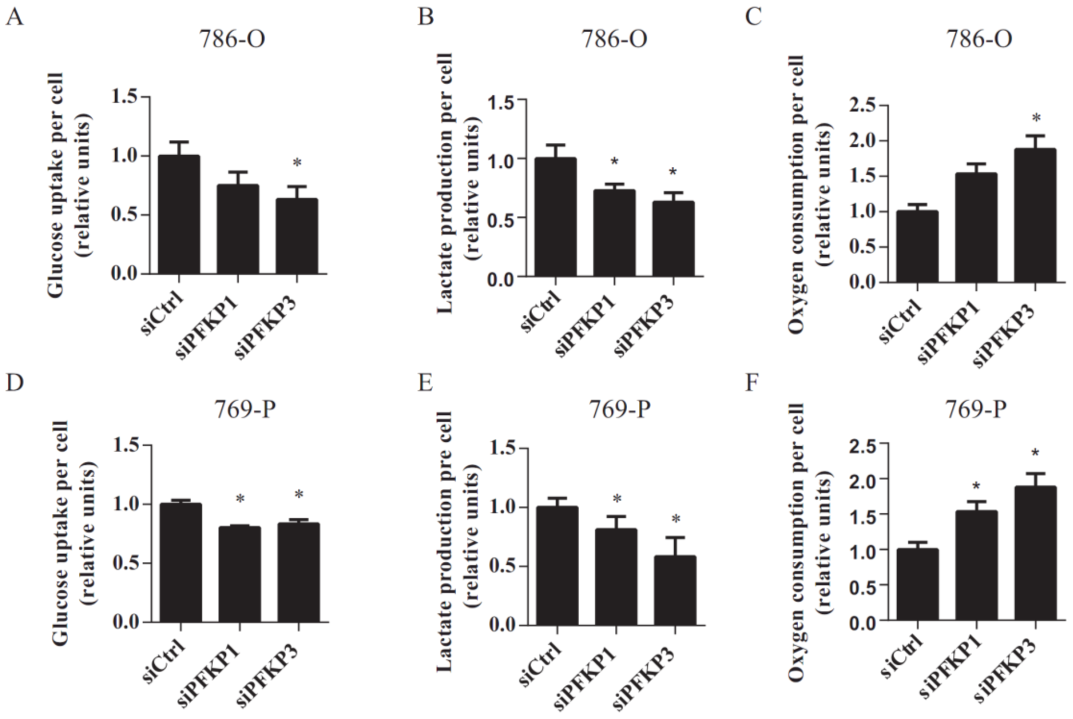

Supplementary Figure S3: Suppression of PFKP decreases glycolysis and activates TCA cycle in kidney cancer cells in 786-O and 769-P cells.

Glucose uptake (A, D), lactate production (B, E) and oxygen consumption rates (C, F) per cell in 786-O and 769-P cells transiently transfected with siCtrl, siPFKP1, or siPFKP3 at 72 hours after transfection.

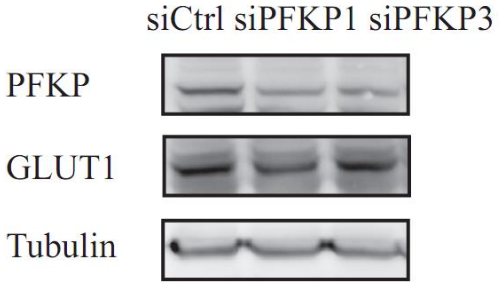

Supplementary Figure S4: Suppression of PFKP does not alter the expression of GLUT1 in Caki-1 cells.

Western blot for protein extracts of Caki-1 cells transiently transfected with siCtrl, siPFKP1 and siPFKP3 at 72 hours after transfection.

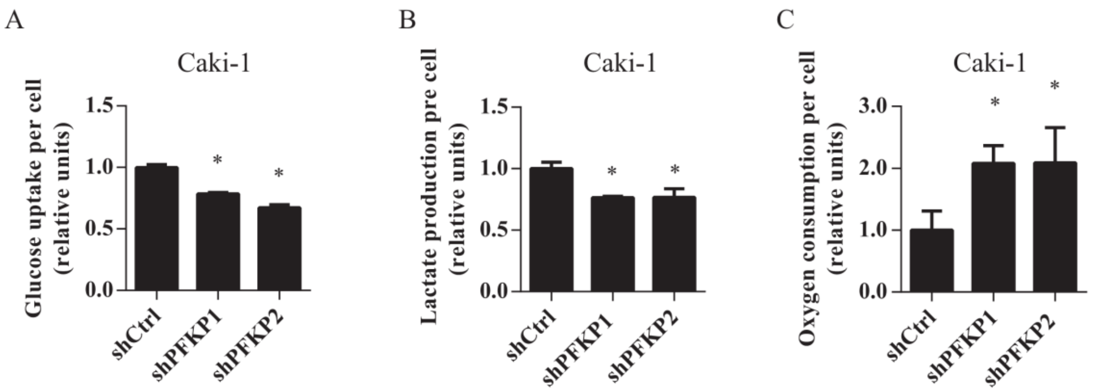

Supplementary Figure S5: Caki-1 cells stably transfected with shPFKP show decreased glycolysis and activated TCA cycle activity.

Glucose uptake (A), lactate production (B) and oxygen consumption rates (C) per cell in Caki-1 cells stably transfected with shCtrl, shPFKP1 or shPFKP2.

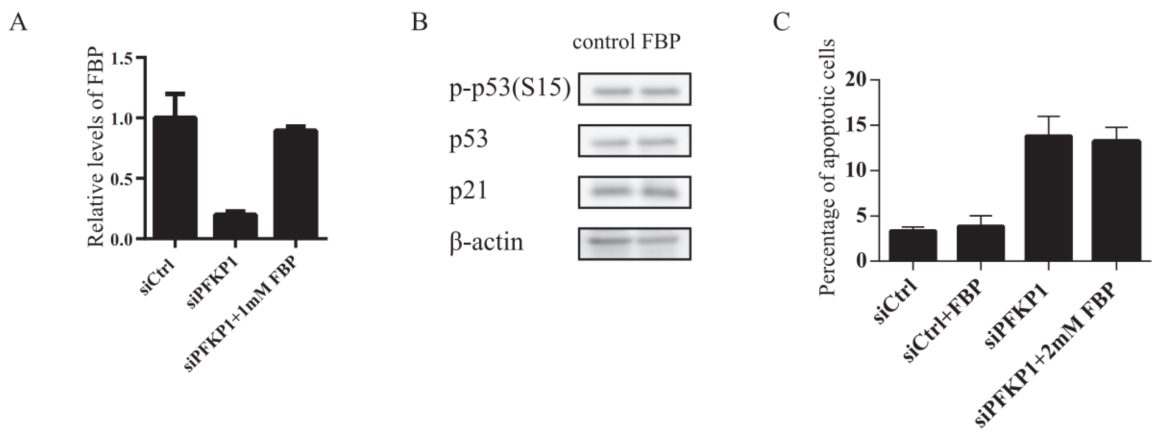

Supplementary Figure S6: FBP treatment increases intracellular FBP levels in Caki-1 cells transiently transfected with siPFKP1.

(A) Intracellular FBP levels in Caki-1 cells transiently transfected with siCtrl or siPFKP1 and treated with 1 mM FBP at 72 hours after transfection. (B) Western blot analysis for Caki-1 cells treated with either vehicle control or 2 mM FBP. (C) Percentage of apoptotic cells in Caki-1 cells transiently transfected with siCtrl or siPFKP1 and treated with 2 mM FBP at 72 hours after transfection.

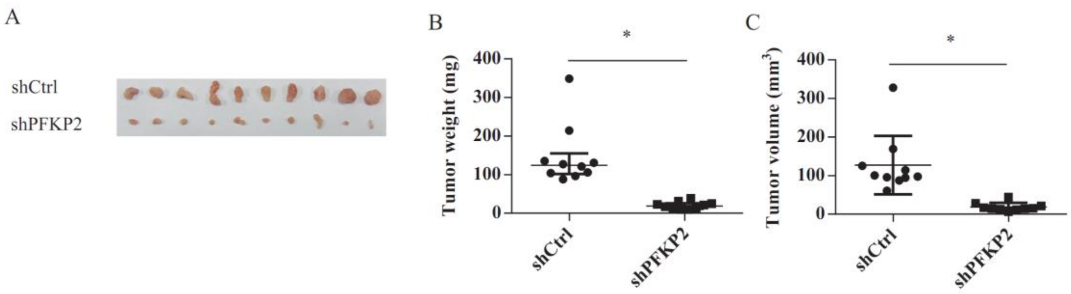

Supplementary Figure S7: PFKP suppression inhibits tumor growth *in vivo*.

(A) Picture of subcutaneous tumor from nude mice injected with Caki-1 cells stably transfected with shCtrl or shPFKP2. (B-C) Weight (B) and size (C) of tumors from nude mice injected with Caki-1 cells stably transfected with shCtrl or shPFKP2 at 30 days after injection (n=10).

Supplementary Table 1. Clinical feature of patients

No.	gender	age	pathology	grade
1	male	61	ccRCC	2
2	female	54	ccRCC	2
4	male	61	ccRCC	2
5	male	46	ccRCC	2
6	male	56	ccRCC	2
7	male	52	ccRCC	3
8	male	61	ccRCC	2
9	female	24	ccRCC	2
10	male	50	ccRCC	2
11	female	71	ccRCC	2
12	female	65	ccRCC	1
13	male	77	ccRCC	2
14	male	49	ccRCC	2
15	male	58	ccRCC	2
17	male	66	ccRCC	2
18	male	62	ccRCC	3
19	female	54	ccRCC	3
20	male	55	ccRCC	2
21	male	74	ccRCC	3

Supplementary Table 2. Q-PCR primer

human β-actin F	GGACTTCGAGCAAGAGATGG
human β-actin R	AGCACTGTGTTGGCGTACAG
human-PFKL-F	CGTGCAGATGACCAAGGAAG
human-PFKL-R	TGGCCAGGGAGAAGTTAGAC
human-PFKP-F	CCCATCACCTCTGAGAAAATC
human-PFKP-R	TTCAGTGACACGACGCAAG
Human-PFKM-F	GGTGCCCGTGTCTTCTTTGT
Human-PFKM-R	AAGCATCATCGAAACGCTCTC
human-PUMA-F	GGAGGGTCCTGTACAATCTC
human-PUMA-R	GCTACATGGTGCAGAGAAAG
human--NOXA-F	ATGAATGCACCTTCACATTCCTCT
human-NOXA-R	TCCAGCAGAGCTGGAAGTCGAGTG
human-p21-F	ACTGTGATGCGCTAATGGC
human-p21-R	ATGGTCTTCCTCTGCTGTCC
human-TIGAR-F	CAGTGATCTCATGAGGACAAAGCA
human-TIGAR-R	CCATGGCCCTCAGCTCACTTA
human-P53-F	CAGCACATGACGGAGGTTGT
human-P53-R	TCATCCAAATACTCCACACGC
